# Supplementary material for: Species-specific modulation of food-search behavior by respiration and chemosensation in Drosophila larvae
Source: eLife. 2017 Sep 5;6:e27057. doi: 10.7554/eLife.27057 (PMC5584988; doi:10.7554/eLife.27057)
Supplement: Transparent reporting form [file elife-27057-transrepform.docx]

***eLife’s* transparent reporting form**

We encourage authors to provide detailed information *within their submission* to facilitate the interpretation and replication of experiments. If you have any questions, please contact us: [editorial@elifesciences.org](mailto:editorial@elifesciences.org).

**Sample-size estimation**

- You should state whether an appropriate sample size was computed when the study was being designed
- You should state the statistical method of sample size computation and any required assumptions
- If no explicit power analysis was used, you should describe how you decided what sample (replicate) size (number) to use

Please outline where this information can be found within the submission (e.g., page numbers or figure legends), or explain why this information doesn’t apply to your submission:

In this work, we chose sample sizes that were sufficiently large to ensure both the significance and the power of comparisons between the means of different samples (i.e., minimization of type I and type II errors). As explained in the “Statistical procedures” section of the Materials and Methods, an initial sample size was estimated from the means and variances obtained from pilot datasets. We then validated the appropriateness of the final sample sizes. For samples that departed from normality, the sample size could not be estimated using a standard method. In those cases, we made a sample-size estimate after assuming the normality of the samples. Normality of the samples was assessed by the Lilliefors test.

**Replicates**

- You should report how often each experiment was performed
- You should include a definition of biological versus technical replication
- The data obtained should be provided and sufficient information should be provided to indicate the number of independent biological and/or technical replicates
- If you encountered any outliers, you should describe how these were handled
- Criteria for exclusion/inclusion of data should be clearly stated
- High-throughput sequence data should be uploaded before submission, with a private link for reviewers provided (these are available from both GEO and ArrayExpress)

Please outline where this information can be found within the submission (e.g., page numbers or figure legends), or explain why this information doesn’t apply to your submission:

The size of every sample of the study are reported in an excel file called “Supplementary file 1”. Since the behavior of a given larva is stochastic and animals could only be tested once, we could not perform technical replications of our experiments. All trials involved animals that behaved independently of each other. As described in the manuscript, our analysis pertains to averages computed over the behavior of different larvae or individual diving sequences. Outliers were reported in boxplots according to the convention described in the figure legends. At the end of each experiment, the behavior of individual larvae was inspected based on a replay of the movies. As explained in the “Materials and Methods”, the minority of larvae that were found to be inactive were excluded from the analysis. These cases account for less than 10% of the trials – inactivity is thought to be due to the mechanical manipulation of the animal prior to their introduction to the assay.

**Statistical reporting**

- Statistical analysis methods should be described and justified
- Raw data should be presented in figures whenever informative to do so (typically when N per group is less than 10)
- For each experiment, you should identify the statistical tests used, exact values of N, definitions of center, methods of multiple test correction, and dispersion and precision measures (e.g., mean, median, SD, SEM, confidence intervals; and, for the major substantive results, a measure of effect size (e.g., Pearson's r, Cohen's d)
- Report exact p-values wherever possible alongside the summary statistics and 95% confidence intervals. These should be reported for all key questions and not only when the p-value is less than 0.05.

The statistical analysis is described in the “Statistical procedures” section of the Materials and Methods. Samples that could not be modeled as normal distributions were represented by boxplots. The statistical tests used throughout the study are reported in the figure legends and the Supplementary File 1. Full information about the sample size and p-values can be found in Supplementary File 1. For Student’s t-test, the Cohen’s *d* values are also reported in Supplementary file 1.

Please outline where this information can be found within the submission (e.g., page numbers or figure legends), or explain why this information doesn’t apply to your submission:

(For large datasets, or papers with a very large number of statistical tests, you may upload a single table file with tests, Ns, etc., with reference to page numbers in the manuscript.)

**Additional data files (“source data”)**

- We encourage you to upload relevant additional data files, such as numerical data that are represented as a graph in a figure, or as a summary table
- Where provided, these should be in the most useful format, and they can be uploaded as “Source data” files linked to a main figure or table
- Include model definition files including the full list of parameters used
- Include code used for data analysis (e.g., R, MatLab)
- Avoid stating that data files are “available upon request”

Please indicate the figures or tables for which source data files have been provided:

Our experiments rely on the quantification of movie sequences. We conducted an in-depth analysis of these movies. We doubt that other experimenters will benefit from accessing raw movies, but we are ready to be convinced otherwise by the reviewers. As we have done with past manuscripts (including one published in eLIFE), we will make an annotated version of the Matlab codes accessible on the Github account of our lab upon acceptance of the manuscript.
